# Survivin-Targeting Antisense Oligonucleotides in Cancer Therapy

**DOI:** 10.3390/molecules31132283

**Published:** 2026-06-30

**Authors:** Bal Hari Poudel, Suxiang Chen, Rakesh N. Veedu

**Affiliations:** 1 Personalised Medicine Centre, Health Futures Institute, Murdoch University, Murdoch, WA 6150, Australia; bal.poudel@murdoch.edu.au; 2 Precision Nucleic Acid Therapeutics, Perron Institute for Neurological and Translational Science, Nedlands, WA 6009, Australia; 3 ProGenis Pharmaceuticals Pty Ltd., Bentley, WA 6102, Australia

**Keywords:** survivin, BIRC5, antisense oligonucleotides, ASO, cancer therapy

## Abstract

Survivin (BIRC5) is a key inhibitor of apoptosis that is highly overexpressed in many cancers, where it promotes tumour cell survival, mitotic progression, and resistance to therapy. Because survivin is largely absent from normal adult tissues, it represents a selective and promising target for cancer treatment. Antisense oligonucleotides (ASOs) provide a precise approach to silence survivin by targeting its transcripts. Preclinical studies have shown that ASO-mediated reduction of survivin is associated with increased cancer cell death, inhibition of tumour growth, and enhanced sensitivity to other treatments. Early-phase clinical trials of survivin-targeting ASOs have shown evidence of target engagement but ultimately failed to demonstrate consistent clinical benefit and/or encountered dose-limiting toxicities, which hindered their further development. This review outlines survivin’s central role in cancer biology, the principles of ASO therapeutics (sequence design, mechanisms of action, chemical modifications, and delivery strategies), and the progress in preclinical and clinical development of survivin-targeting ASOs, while also discussing key challenges that may contribute to their clinical limitations, including inefficient delivery, off-target effects, and systemic toxicities. Collectively, the current status of survivin-targeting ASOs underscores the need for synergistic optimization of delivery platforms and molecular chemistry to improve efficacy and safety, thereby enabling their use in personalised and combination cancer treatment approaches.

## 1. Introduction

Malignant transformation is characterised by capabilities acquired by cancer cells, including evasion of cell death, sustained proliferative signalling, and genomic instability and mutation [1]. Regulatory proteins often coordinate these capabilities by integrating cell division with survival pathways. Survivin (baculoviral inhibitor of apoptosis repeat-containing 5, BIRC5) is a key component of the chromosomal passenger complex (CPC) that regulates essential mitotic processes such as chromosome segregation and cytokinesis, while also promoting cell survival under stress conditions [2].

In normal physiology, survivin expression is low or undetectable in most differentiated adult tissues, although it is retained in certain proliferative cell populations. In contrast, survivin is frequently upregulated in a wide range of human tumours, including lung and breast cancers, where its expression has been associated with increased aggressiveness, resistance to therapy, and poor clinical outcomes [2,3]. This tumour-selective expression pattern, together with its dual role in mitosis and cell survival, has generated significant interest in survivin as both a therapeutic target and a prognostic indicator.

Antisense oligonucleotides (ASOs) offer a strategy for selectively reducing gene expression at the RNA level. These molecules exert their effects by hybridising to complementary RNA sequences, leading either to RNase H-mediated transcript degradation or to steric blocking of RNA function, which can alter processes such as splicing or translation [4,5]. Chemical modifications have improved the stability and pharmacokinetic properties of ASOs [6], thereby enhancing their capacity to reduce target RNA levels in preclinical settings.

This review summarises the biological functions of survivin in cancer and outlines the principles of ASO-based therapeutics, including antisense sequence design, mechanisms of action, chemical modifications, and delivery strategies. It further discusses current progress in the development of survivin-targeting ASOs and identifies critical challenges that may influence their clinical translation, such as delivery barriers, off-target effects, and systemic toxicities. Finally, it highlights future design strategies aimed at expanding the therapeutic window.

## 2. Survivin (BIRC5): Gene, Protein Structure, and Regulation

*BIRC5* is located on chromosome 17q25 and is transcribed from a TATA-less promoter containing multiple specificity protein 1 (Sp1)-binding sites and cell cycle regulatory elements (cell cycle-dependent element [CDE]/cell cycle genes homology region [CHR]), with these components together coordinating cell cycle-dependent transcriptional control during G2/M phase progression [7]. This promoter organization underlies the tightly regulated, cell cycle–restricted expression pattern of *BIRC5* rather than constitutive transcription. *BIRC5* expression is regulated by multiple signaling pathways, including p53, Wnt/β-catenin, and Notch, which influence *BIRC5* transcription through tumor suppressor- and oncogene-associated regulatory networks [8].

Survivin is a 142 amino acid inhibitor of apoptosis protein (IAP) family protein containing a single baculoviral IAP repeat (BIR) domain and a C-terminal α-helical region, which contributes to homodimer formation [9]. Within the CPC, survivin interacts with core components, including borealin and inner centromere protein (INCENP), to form a stable heterotrimeric assembly [10]. In this context, survivin directly recognizes phosphorylated histone H3 at threonine 3 (H3T3ph), a histone modification that serves as a chromatin-based docking signal required for CPC centromere targeting and subsequent Aurora B kinase activation [11].

*BIRC5* pre-mRNA undergoes alternative splicing to generate multiple transcript variants, such as survivin-ΔEx3, survivin-2B, and survivin-2α [12,13]. These splice variants are generally expressed at lower levels relative to the canonical full-length isoform, although their expression may vary across different tissue types and disease contexts [14,15]. Survivin-ΔEx3 retains anti-apoptotic activity, whereas survivin-2B contains an insertion that disrupts the BIR domain and is associated with reduced anti-apoptotic potential [12]. Survivin-2α represents a truncated isoform with limited functional characterization at the protein level [13]. Clinical studies have reported associations between survivin splice variant expression patterns and clinical outcomes in hematological and solid malignancies [16,17].

Post-translational regulation of survivin involves multiple modifications, including phosphorylation, acetylation, and ubiquitination, which govern its functional activity, intracellular localization, and protein stability. Phosphorylation of survivin at threonine 34 by cyclin-dependent kinase 1 (CDK1) is critical for its anti-apoptotic function, whereas Aurora B-mediated phosphorylation modulates its role during mitosis [18,19]. Acetylation of survivin has been shown to promote its nuclear accumulation and repress signal transducer and activator of transcription 3 (STAT3)-dependent transcriptional activity, linking post-translational modification to transcriptional regulation [20]. In addition, ubiquitination-dependent turnover of survivin is tightly controlled by deubiquitinases such as USP35, which stabilize the protein and sustain its cellular functions [21]. Survivin also undergoes chromosome region maintenance 1 (CRM1, also known as exportin 1 [XPO1])-dependent nuclear export, a process that determines its predominant cytoplasmic localization and thereby sustains its anti-apoptotic activity [22]. Furthermore, a mitochondrial pool of survivin has been identified in cancer cells, where it directly inhibits apoptosis and promotes tumorigenesis, although the precise regulatory mechanisms underlying its mitochondrial function remain incompletely defined [23].

## 3. Biological Role of Survivin in Cancer

### 3.1. Anti-Apoptotic Functions

**Figure 1:**
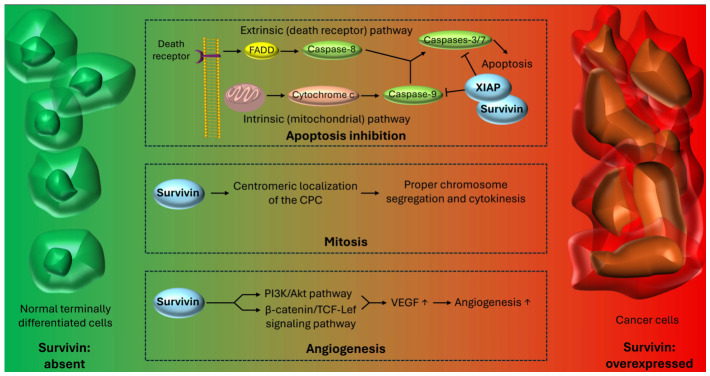
Overall role of survivin in apoptosis, mitosis and angiogenesis. FADD: Fas-associated death domain, XIAP: X-linked inhibitor of apoptosis protein, CPC: chromosomal passenger complex, PI3K: phosphatidylinositol 3-kinase, Akt: protein kinase B, VEGF: vascular endothelial growth factor.

Survivin plays a central role in inhibiting apoptosis, the programmed cell death essential for maintaining tissue homeostasis and eliminating damaged or dangerous cells [24,25]. It functions through multiple mechanisms, primarily by indirectly modulating caspase activity, particularly caspase-3 and caspase-7, via interactions with other IAP family members, and by stabilising XIAP (X-linked inhibitor of apoptosis protein), thereby enhancing cell survival pathways [3,25]. Additionally, survivin has been reported to associate with mitochondrial pathways involved in apoptosis and forms complexes with other IAP proteins, contributing to resistance against intrinsic apoptosis triggered by cellular stress or DNA damage. Its presence in the cytoplasm, mitochondria, and nucleus underlines its multifaceted role in regulating cell death pathways [25]. High survivin expression is often associated with resistance to chemotherapy and radiotherapy, as it enables cancer cells to evade apoptosis. Conversely, survivin silencing can sensitise tumour cells to therapy and partially restore apoptotic responses [3,25]. Overall, the diverse roles of survivin are summarised in Figure 1.

### 3.2. Role in Mitosis

Survivin is critical in regulating mitosis. It is a core component of the CPC, which includes Aurora B kinase, INCENP, and Borealin, and is essential for accurate chromosome alignment, segregation, and cytokinesis. Survivin dynamically localises during mitosis, associating with centromeres in early stages and redistributing to the spindle midzone during later phases. Dysregulation of survivin expression can disrupt this tightly controlled localisation, impairing chromosome segregation and mitotic progression. Consequently, cells may develop chromosomal instability, a hallmark of cancer associated with tumour heterogeneity and aggressiveness. These mitotic roles are supported by multiple studies, including those demonstrating that altered survivin levels are associated with chromosomal instability and mitotic defects in tumour cells [24,26,27].

### 3.3. Role in Angiogenesis

Survivin also plays an important role in tumour angiogenesis. Accumulating evidence suggests that survivin is associated with enhanced vascular endothelial growth factor (VEGF) signalling and VEGF-dependent angiogenic activity, at least in part through activation of the phosphatidylinositol 3-kinase/protein kinase B (PI3K/Akt) and β-catenin/TCF-Lef signalling pathways [28]. These pro-angiogenic effects are supported by functional studies showing that modulation of survivin expression alters neovascularisation in both in vitro and in vivo models, including the chick chorioallantoic membrane (CAM) assay [29,30].

## 4. ASO Therapeutics

### 4.1. ASO Sequence Design

ASO activity is strongly dependent on the position of the binding site along the target RNA [31]. Although thermodynamic parameters and structural accessibility criteria are critical for identifying functional antisense sequences [32], predicting ASO efficacy in live cells from cell-free biophysical models alone remains a significant challenge, motivating the use of advanced computational frameworks [33]. One common design strategy is to generate large numbers of overlapping, fixed-length antisense sequences to cover the full length of the target RNA, which can be implemented computationally by systematic sequence enumeration [34]. This results in a large pool of candidate sequences that must be screened to identify the most potent ones and exclude those with potential off-target toxicity. Specifically, computer-aided drug design (CADD) and machine learning (ML) frameworks are used to rank candidate sequences based on their predicted antisense potency [33,35]. Concurrently, transcriptome-wide alignments are deployed to detect potential cross-hybridization risks [33,34], which provide a basis for filtering out candidates with elevated safety liabilities [33]. Through this performance-guided computational pipeline, thousands of raw candidate sequences are reduced to a highly curated, manageable panel for subsequent chemical synthesis and experimental validation.

### 4.2. Mechanism of Action

**Figure 2:**
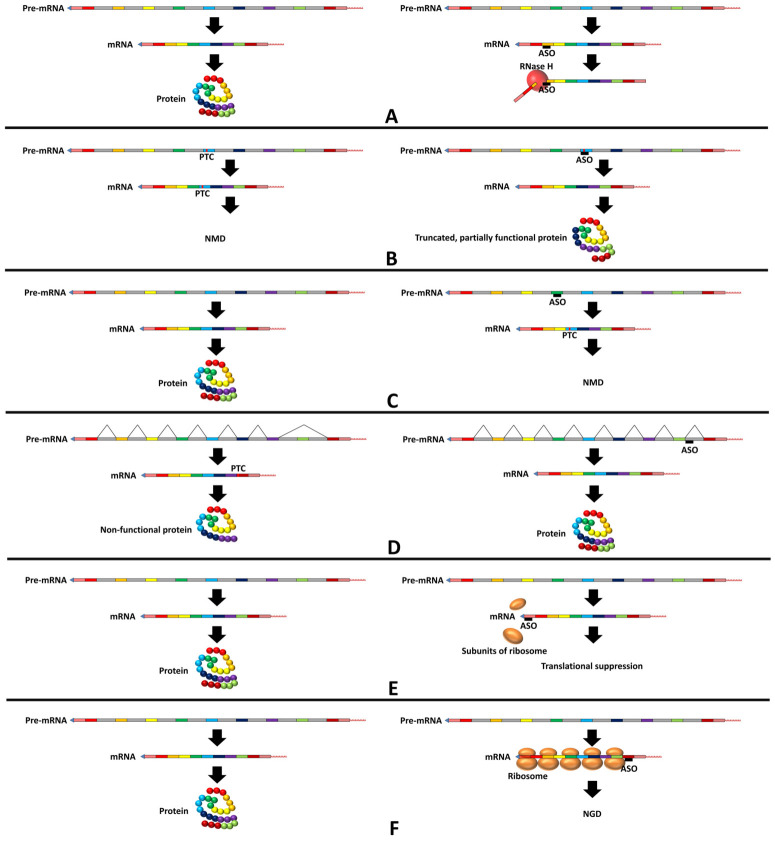
Mechanisms of action of ASOs. (**A**) RNase H-mediated cleavage of the ASO/RNA heteroduplex. (**B**) Exon skipping to restore the open reading frame by removing a PTC-containing exon. (**C**) Exon skipping to induce a frameshift and downstream PTC, triggering NMD. (**D**) Exon inclusion to restore the open reading frame, thereby increasing full-length protein production. (**E**) Translation suppression via steric hindrance of translation initiation complex assembly. (**F**) Ribosome stalling on translating mRNA, triggering NGD. ASO: antisense oligonucleotide, PTC: premature termination codon, NMD: nonsense-mediated mRNA decay, NGD: no-go decay.

ASOs alter gene expression through two major mechanisms of action: RNA cleavage and steric blockage [36,37] (Figure 2). The cleavage pathway is mediated by endogenous RNase H1, which recognizes the DNA/RNA heteroduplex formed between the ASO and its target RNA and catalyzes site-specific cleavage of the RNA strand, which is subsequently degraded by cellular RNA decay machinery [38]. In contrast, the steric-blockage mechanism does not induce direct enzymatic cleavage; instead, the bound ASO physically interferes with molecular interactions on the target transcript [39]. A major application of this steric hindrance is splice switching, which includes both exon skipping and exon inclusion to modulate gene expression. Exon skipping can either restore an open reading frame by removing an exon containing a premature termination codon (PTC) [40] or downregulate gene expression by inducing a frameshift that generates a downstream PTC, thereby triggering nonsense-mediated mRNA decay (NMD) [41]. In addition, exon inclusion is exemplified by nusinersen, which promotes the inclusion of exon-7 during *SMN2* pre-mRNA splicing, resulting in increased production of functional full-length SMN protein [42]. Beyond splicing regulation, steric-blocking ASOs can interfere with the formation of the translation initiation complex, thereby inhibiting protein translation [43], and can also induce ribosome stalling on translating mRNAs, leading to mRNA reduction through no-go decay (NGD)-like mRNA quality control pathways [44].

### 4.3. Chemical Modifications

Unmodified oligonucleotides are rapidly degraded in biological fluids, which limits their clinical utility. To improve their therapeutic performance, ASOs are chemically modified to enhance stability, increase binding affinity to target RNA, and reduce degradation by nucleases. Several backbone and sugar modifications have been developed and are now widely used in ASO design [45]. Crucially, the choice of these chemical modifications to some extent determines the mechanism of action of the ASO. While certain modifications maintain the ability of the ASO to recruit RNase H for target RNA cleavage, others fail to support this recruitment, restricting the ASO to a steric hindrance mechanism.

**Figure 3:**
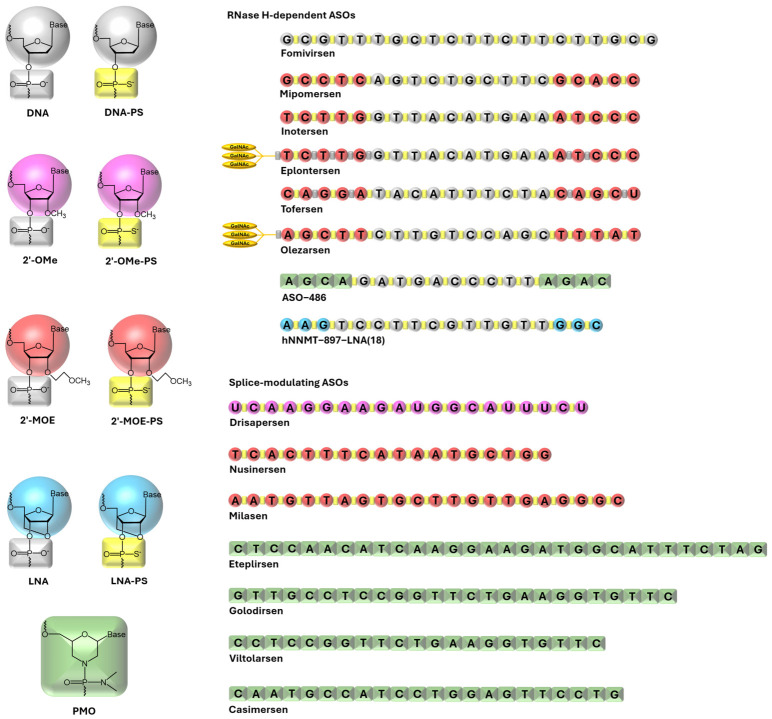
Examples of nucleotide analogues and chemically modified ASOs. ASO: antisense oligonucleotide, PS: phosphorothioate, 2′-OMe: 2′-O-methyl, 2′-MOE: 2′-O-methoxyethyl, LNA: locked nucleic acid, PMO: phosphorodiamidate morpholino oligomer, GalNAc: N-Acetylgalactosamine. ASO−486 was reported by Kanatsu et al. (2024) [57], hNNMT−897−LNA(18) was reported by Hara et al. (2025) [55].

The most common inter-nucleotide phosphate linkage modification is phosphorothioate (PS) [46], where a non-bridging oxygen atom in the phosphate group is replaced by a sulfur atom (Figure 3). This seemingly small change significantly increases resistance to nuclease degradation and improves plasma protein binding, which in turn prolongs circulation time in vivo. However, PS modification can also lead to non-specific protein interactions, which may contribute to sequence-independent toxic effects [47]. Driven by the need to reduce PS abundance, recent studies have focused on modulating backbone composition (phosphodiester [PO]/PS mixmers) and optimizing the positioning of the remaining PS linkages [48,49].

Another important class of modifications involves altering the sugar moiety of the nucleotide (Figure 3). For example, 2′-O-methyl (2′-OMe) and 2′-O-methoxyethyl (2′-MOE) modifications increase the binding affinity of ASOs to their target RNA by enhancing base stacking and duplex stability [50]. These modifications also improve resistance to nucleases and extend the half-life of ASOs in biological systems. In addition, they can reduce immunostimulatory activity [51], which is a common concern with oligonucleotide therapeutics. The 2′-MOE modification, in particular, is widely used in clinically approved ASOs due to its favourable balance between stability, potency, and safety. Locked nucleic acid (LNA) represents another powerful sugar modification, in which the ribose ring is chemically constrained into a fixed conformation [52]. This “locking” significantly enhances hybridisation affinity for complementary RNA sequences. As a result, LNA-containing ASOs can achieve strong target binding even at shorter lengths [53], which may reduce dosing requirements. More structurally distinct chemistries include phosphorodiamidate morpholino oligomers (PMOs), which replace the natural ribose sugar with a morpholine ring and use a phosphorodiamidate linkage instead of the standard phosphodiester backbone (Figure 3). This neutral backbone makes PMOs highly resistant to enzymatic degradation and largely eliminates interactions with nucleases and many proteins [54].

Importantly, while PS linkages preserve the capacity of ASOs to recruit RNase H, sugar modifications (such as 2′-OMe, 2′-MOE, and LNA) and fully altered backbones (such as PMOs) disable this capability. To exploit the RNase H-based mechanism without sacrificing nuclease stability and high target-binding affinity, the “gapmer” design was developed. A typical gapmer features a central DNA region (often carrying a fully PS-modified backbone) capable of recruiting RNase H, flanked by sugar-modified nucleotides. This architecture has been widely translated into clinical applications. For instance, the MOE–DNA–MOE gapmer modality has been adopted for multiple approved antisense therapeutics, including mipomersen, inotersen, volanesorsen, tofersen, eplontersen, and olezarsen. In addition, LNA-based gapmers are also being evaluated; a recently reported LNA gapmer, hNNMT-897-LNA(18), demonstrated robust efficacy in downregulating nicotinamide N-methyltransferase (NNMT) [55,56]. In contrast, uniformly modified PMOs have often been employed for steric-blocking purposes, as demonstrated by multiple splice-switching drugs (eteplirsen, golodirsen, viltolarsen, casimersen) approved for treating Duchenne muscular dystrophy (DMD). A recent study has also adopted a PMO–DNA–PMO gapmer design to recruit RNase H for target degradation [57,58].

### 4.4. Delivery Strategies

**Figure 4:**
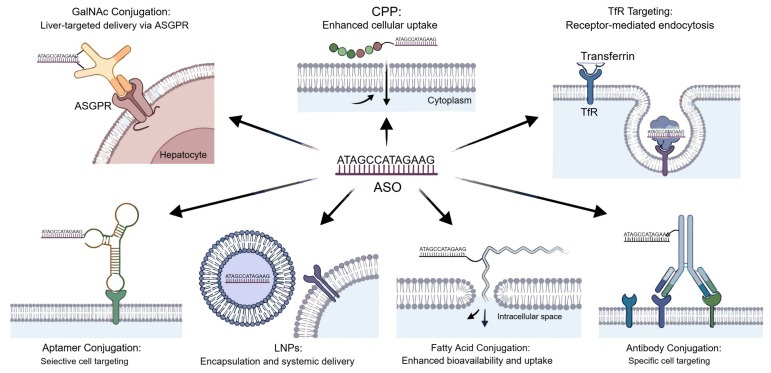
Image showing different methods used to improve ASO drug delivery. The figure was created using Biorender. ASGPR: Asialoglycoprotein receptor, TfR: transferrin receptor, GalNAc: N-Acetylgalactosamine, LNPs: lipid nanoparticles, CPP: cell penetrating peptide, ASO: antisense oligonucleotide.

Effective delivery remains one of the most significant barriers to the clinical success of ASOs. Although ASOs can be chemically stabilised, their efficient transport across biological membranes, avoidance of rapid clearance, and accumulation within target tissues—particularly solid tumours—are still challenging. Multiple delivery strategies are therefore being developed to improve cellular uptake, biodistribution, and target specificity [59]. Different strategies for efficient ASO drug delivery are presented in Figure 4.

Lipid nanoparticles (LNPs) have emerged as one of the most promising delivery platforms. These systems encapsulate ASOs within lipid-based vesicles, protecting them from degradation and facilitating cellular uptake through endocytosis. The success of LNPs in mRNA vaccine technologies has accelerated their adaptation for ASO delivery [60,61]. Importantly, LNP composition can be tuned to optimise tissue distribution and endosomal escape, which is critical for releasing ASOs into the cytoplasm where they can access their target RNA.

Other methods include conjugation with ligands such as N-acetylgalactosamine (GalNAc) for targeted liver delivery [62], use of cell-penetrating peptides (CPPs) [63,64], and antibody–oligonucleotide conjugates (AOCs) for receptor-mediated uptake [65]. In addition to these approaches, conjugation strategies have broadened to encompass aptamers or lipophilic moieties such as fatty acids and vitamin E [66,67], which are commonly integrated with bio-responsive linkers to facilitate cellular cargo release [68]. Ultimately, translating these diverse platforms into survivin-targeted cancer therapies relies on exploiting intracellular trafficking pathways, as exemplified by the discovery that the endosomal maturation protein WDR91 is required to promote productive ASO release and potency within tumor cells [69,70].

## 5. ASOs Targeting Survivin

### 5.1. Preclinical Studies

To the best of our knowledge, six independently developed ASO sequences targeting survivin have been reported to date, including ISIS 23722 (LY2181308) [26,71,72,73,74,75,76,77,78,79], SPC3042 (EZN-3042) [75,80,81,82,83,84], oligonucleotide 4003 [85,86,87], aODN-Surv [88], an ASO targeting survivin developed by Sun et al. [89], and BIRC5 H2A (+86+110) [90] (Table S1).

Oligonucleotide 4003, one of the early PS-modified DNA ASOs targeting survivin, was reported to induce apoptosis and enhance chemosensitivity in lung cancer cells, including the A549 cell line [85]. It also inhibited proliferation and induced apoptosis in hepatocellular carcinoma and osteosarcoma cells [86,87]. Similarly, another PS-modified DNA ASO targeting survivin was shown to suppress tumor growth in an orthotopic nude mouse model of human hepatocellular carcinoma [89]. In addition, a DNA-based ASO with a mixed PO/PS backbone (aODN-Surv) was evaluated in tumor-associated angiogenesis-related assays, where it was reported to affect endothelial cell proliferation, migration, and tube formation [88].

ISIS 23722 (also referred to as LY2181308), an MOE–DNA–MOE gapmer with a full PS backbone, has been investigated in multiple hematological and solid tumor models. A variant of ISIS 23722 with a mixed PO/PS backbone (ISIS 28599) demonstrated that survivin downregulation leads to apoptosis, defective cytokinesis, and loss of anchorage-independent growth in cancer cell models [79]. Additional studies showed that survivin suppression resulting from ISIS 23722 treatment induces cell division defects and apoptosis [26] and activates mitochondrial apoptotic pathways in myeloid leukemia cells [71]. In aggressive non-Hodgkin’s lymphoma, ISIS 23722 suppressed tumor cell proliferation [74]. In cultured lung cancer cells, ISIS 23722 enhanced radiosensitivity and increased cytotoxic effects following irradiation [72,73], while in vivo studies demonstrated reduced tumor growth in xenograft models of lung cancer and other solid tumors [76,77]. A more recent study developed an NAD(P)H oxidoreductase 1 (NQO1)-activatable circular ISIS 23722, in which enzymatic reduction in tumor cells triggers conversion to an active linear form, thereby enabling tumor-selective activation and enhanced antitumor efficacy [78].

SPC3042 (also referred to as EZN-3042), an LNA-modified gapmer with a PS backbone, has also been evaluated in multiple cancer models. An initial study demonstrated its potent proapoptotic activity and compared it with ISIS 23722 under different chemical configurations [75]. Further studies have shown that SPC3042 induces apoptosis via mitotic catastrophe in neuroblastoma cells [81], overcomes drug resistance in acute lymphoblastic leukemia [82], and enhances chemotherapeutic responses in leukemia models [83]. While in vivo efficacy was primarily established in tumor xenograft models, including lung cancer-derived systems [80], the translational potential of SPC3042 was further demonstrated in a veterinary phase-I trial through successful survivin knockdown in dogs with spontaneous lymphoma [84].

In contrast to the aforementioned RNase H-dependent ASOs, BIRC5 H2A (+86+110) represents a fully 2′-OMe-PS ASO that functions as a steric blocker. In HepG2 cells, this ASO reduced survivin expression at the mRNA and protein levels [90].

### 5.2. Clinical Trials

LY2181308 and EZN-3042 are two survivin-targeting ASOs that have undergone clinical evaluation. Preclinical pharmacokinetic, toxicological, and exposure–response analyses informed dose selection and the design of the first-in-human study of LY2181308 [91]. In that study, intravenous administration was associated with reductions in survivin mRNA and protein expression in paired tumour biopsy samples in some patients [92]. A subsequent Phase I study in Japanese patients with advanced solid tumours assessed the pharmacodynamic activity of LY2181308 in peripheral blood mononuclear cells, where modulation of *BIRC5* mRNA expression was observed in a proportion of patients, indicating interpatient variability [93]. In another Phase I study in patients with relapsed or refractory acute myeloid leukemia, LY2181308 was administered as monotherapy or in combination with idarubicin and cytarabine [94]. The drug exhibited linear pharmacokinetics and was generally well tolerated within the tested dose range. However, dose-limiting hematologic toxicities were reported, and anti-leukemic activity was modest in this study.

In Phase II studies, LY2181308 was evaluated in combination with standard chemotherapy regimens. In metastatic castration-resistant prostate cancer, the addition of LY2181308 to docetaxel and prednisone did not improve progression-free or overall survival compared with chemotherapy alone and was associated with increased hematologic toxicity, including neutropenia [95]. Similarly, when LY2181308 was combined with docetaxel, a randomized Phase II study in non-small-cell lung cancer reported no improvement in tumor response or clinical outcomes [96]. In addition, a case report described reversible renal dysfunction in a patient with metastatic melanoma during prolonged treatment with LY2181308, in which elevated serum creatinine levels returned to baseline following drug discontinuation [97].

EZN-3042 was evaluated in a Phase I pediatric study in patients with relapsed acute lymphoblastic leukemia in combination with intensive re-induction chemotherapy [98]. Pharmacodynamic evidence of reduced survivin expression was observed in peripheral blood samples. However, dose-limiting toxicities, including elevated hepatic enzymes and gastrointestinal hemorrhage, were reported, which prevented completion of dose escalation and determination of the maximum tolerated dose.

## 6. Clinical Translation Challenges

### 6.1. Clinical Lessons from Survivin-Targeting ASOs

Despite promising preclinical findings, survivin-targeting ASOs have not demonstrated meaningful therapeutic benefit in clinical settings. LY2181308 has been reported to reduce survivin mRNA and protein levels in peripheral blood mononuclear cells of evaluable patients and, occasionally, in tumor biopsies [92]; however, these effects were generally modest and inconsistently observed [93,94], and did not translate into improved outcomes in Phase II studies [95,96]. EZN-3042 was associated with dose-limiting toxicities that restricted its clinical evaluation, with limited reported pharmacodynamic data on survivin modulation [98]. Collectively, these findings suggest that insufficient and inconsistent suppression of survivin at tolerated dose levels, together with the safety constraints, likely contributed to the lack of clinical efficacy observed.

### 6.2. Delivery Barriers and Tumor Pharmacology

Efficient delivery of ASOs to tumor cells remains a major challenge, as systemically administered ASOs are known to preferentially accumulate in the liver and kidney, resulting in limited tumor exposure [99,100]. In addition, intracellular trafficking barriers, particularly endosomal sequestration, can restrict the release of ASOs into the cytosol and nucleus where target RNA resides [99,100,101]. These limitations may reduce the effective intracellular concentration of survivin-targeting ASOs and thus weaken target engagement. Current delivery strategies, including ligand-directed conjugates, nanoparticle-based delivery systems, and stimuli-responsive carriers, aim to enhance tumor targeting and promote intracellular release [66,101], and improving endosomal escape is increasingly recognized as an important determinant of ASO efficacy [100].

### 6.3. Safety Liabilities and Off-Target Effects

The safety profile of ASOs is influenced by both sequence and chemical modifications. ASOs can exhibit hybridization-mediated off-target effects due to partial complementarity with non-target transcripts [102,103]. In the case of gapmer ASOs, such interactions may result in unintended cleavage of partially complementary RNAs through RNase H recruitment [103]. Concurrently, PS-modified backbones promote nonspecific protein binding, and both backbone chemistry and nucleotide sequence contribute to differences in protein–ASO interactions, which have been associated with hepatotoxicity, thrombocytopenia, and immune stimulation in a sequence-dependent manner [99,104]. Clinical experience with EZN-3042 exemplifies the challenges associated with LNA-modified ASOs, suggesting that LNA-imparted high hybridization affinity can heighten off-target risks, while PS-mediated nonspecific protein interactions potentially drive systemic toxicities, thereby collectively limiting the therapeutic window [105,106,107].

### 6.4. Future Design Strategies

Future development of survivin-targeting ASOs will likely require coordinated improvements in delivery efficiency and molecular design. Advanced delivery platforms may enhance systemic biodistribution, tumor accumulation, and intracellular availability. At the chemical level, incorporating alternative backbone or sugar modifications and/or optimizing the positioning of different modifications to fine-tune binding affinity, combined with avoiding known toxic sequence motifs, could be a viable approach to mitigating off-target effects and systemic toxicities, thereby expanding the therapeutic window.

## 7. Conclusions

Multiple efforts have been devoted to developing ASOs targeting survivin, given its critical roles in tumor cell survival, mitotic regulation, and chemoresistance. However, clinical translation has largely stalled following unsuccessful human trials, where only limited and inconsistent reductions in survivin expression were observed, together with dose-limiting toxicities. These outcomes are most plausibly attributed to inadequate target engagement in tumors, driven by inefficient delivery, as well as off-target effects and systemic toxicities associated with ASO chemistry. Future progress in this area will likely depend on revisiting previously developed lead ASO sequences and using them as structural templates to systematically optimize chemical modifications for improved safety profiles. In parallel, integrating these sequences into emerging delivery platforms may enhance tumor exposure and intracellular availability, thereby increasing the likelihood of achieving therapeutically meaningful survivin suppression.

## Data Availability

Not applicable.

## Supplementary Materials

The following supporting information can be downloaded at: https://www.mdpi.com/article/10.3390/molecules31132283/s1, Table S1: Chemically modified ASO-based survivin inhibitors developed as research tools and/or anti-cancer therapeutics [26,71,72,73,74,75,76,77,78,79,80,81,82,83,84,85,86,87,88,89,90,92,93,94,95,96,98].

## Abbreviations

The following abbreviations are used in this manuscript:

Akt	Protein kinase B
AOC	Antibody–oligonucleotide conjugate
ASGPR	Asialoglycoprotein receptor
ASO	Antisense oligonucleotide
BIRC5	Baculoviral inhibitor of apoptosis repeat-containing 5
CAM	Chick chorioallantoic membrane
CADD	Computer-aided drug design
CDK1	Cyclin-dependent kinase 1
CHR	Cell cycle genes homology region
CDE	Cell cycle-dependent element
CPC	Chromosomal Passenger Complex
CPP	Cell-penetrating peptide
CRM1	Chromosome region maintenance 1
DMD	Duchenne muscular dystrophy
FADD	Fas-associated death domain
GalNAc	N-Acetylgalactosamine
H3T3ph	Histone H3 Thr-3 phosphorylation
IAP	Inhibitor of apoptosis proteins
INCENP	Inner centromere protein
LNA	Locked nucleic acid
LNP	Lipid nanoparticle
ML	Machine learning
NGD	No-go decay
NMD	Nonsense-mediated mRNA decay
NNMT	Nicotinamide N-methyltransferase
NQO1	NAD(P)H oxidoreductase 1
PI3K	Phosphatidylinositol 3-kinase
PMO	Phosphorodiamidate morpholino oligomer
PO	Phosphodiester
PS	Phosphorothioate
PTC	Premature termination codon
Sp1	Specificity protein 1
STAT3	Signal transducer and activator of transcription 3
TfR	Transferrin receptor
VEGF	Vascular endothelial growth factor
XIAP	X-linked inhibitor of apoptosis protein
XPO1	Exportin 1
2′-MOE	2′-O-methoxyethyl
2′-Ome	2′-O-methyl
