# Supplementary material for: Survivin-Targeting Antisense Oligonucleotides in Cancer Therapy
Source: Molecules. 2026 Jun 30;31(13):2283. doi: 10.3390/molecules31132283 (PMC13363050; doi:10.3390/molecules31132283)
Supplement: Supplementary file 1 [file molecules-31-02283-s001.zip › molecules-4288324-supplementary.pdf]

# Supplementary Information

| No. | ASO name | ASO<br>sequence & chemistry | Ref. | Mechanisms |
|---|---|---|---|---|
| 1 | ISIS 23722<br>(also known as<br>LY2181308) | 18mer 4–10–4 MOE$^{PS}$–DNA$^{PS}$–MOE$^{PS}$ gapmer<br>T*G*T*G*C*T*A*T*T*C*T*G*T*G*A*A*T*T | 1–14 | RNase H-<br>dependent<br>mRNA<br>degradation |
| | | 18mer 4–10–4 MOE$^{PO}$–DNA$^{PS}$–MOE$^{PO}$ gapmer<br>T*G*T*G*C*T*A*T*T*C*T*G*T*G*A*A*T*T | 15 | |
| | | 18mer 4–10–4 LNA$^{PS}$–DNA$^{PS}$–LNA$^{PS}$ gapmer<br>T*G*T*G*C*T*A*T*T*C*T*G*T*G*A*A*T*T | 6 | |
| | | 18mer DNA$^{PS}$<br>T*G*T*G*C*T*A*T*T*C*T*G*T*G*A*A*T*T | 6 | |
| 2 | SPC3042<br>(also known as<br>EZN-3042) | 16mer 4–8–3–1 LNA$^{PS}$–DNA$^{PS}$–LNA$^{PS}$–DNA gapmer<br>C*T*C*A*A*T*C*C*A*T*G*G*C*A*G*C | 6,16–21 | |
| | | 16mer 4–8–3–1 MOE$^{PS}$–DNA$^{PS}$–MOE$^{PS}$–DNA gapmer<br>C*T*C*A*A*T*C*C*A*T*G*G*C*A*G*C | 6 | |
| 3 | Oligonucleotide<br>4003 | 20mer DNA$^{PS}$<br>C*C*C*A*G*C*C*T*T*C*C*A*G*C*T*C*C*T*T*G | 22–24 | |
| 4 | aODN-Surv | 21mer DNA$^{PO-PS}$<br>G*G*G*C*A*A*C*G*T*C*G*G*G*G*C*A*C*C*C*A*T | 25 | |
| 5 | ASO against<br>survivin | 20mer DNA$^{PS}$<br>A*C*C*C*A*T*G*C*C*G*C*C*G*C*C*G*C*C*A*C | 26 | |
| 6 | BIRC5 H2A<br>(+86+110) | 25mer 2'-OMe$^{PS}$<br>A*U*G*G*G*G*U*C*G*U*C*A*U*C*U*G*G*C*U*C*C*C*A*G*C | 27 | Steric blocking |

**Table S1.** Chemically modified ASO-based survivin inhibitors developed as research tools and/or anti-cancer therapeutics. Red stars "*" in ASO sequences represent PS modified linkages, while black stars represent natural PO linkages. Black color represents deoxyribonucleotide, blue color represents 2'-MOE analogue, green color represents LNA analogue, and purple color represents 2'-OMe analogue. ASO: antisense oligonucleotide, PS: phosphorothioate, PO: phosphodiester, 2'-MOE: 2'-O-methoxyethyl, LNA: locked nucleic acid, 2'-OMe: 2'-O-methyl.

[OFFICIAL]

## References


1. Li, F.; Ackermann, E.J.; Bennett, C.F.; Rothermel, A.L.; Plescia, J.; Tognin, S.; Villa, A.; Marchisio, P.C.; Altieri, D.C. Pleiotropic cell-division defects and apoptosis induced by interference with survivin function. *Nat Cell Biol*. **1999**, 1, 461–466.

2. Carter, B.Z.; Wang, R.Y.; Schober, W.D.; Milella, M.; Chism, D.; Andreeff, M. Targeting survivin expression induces cell proliferation defect and subsequent cell death involving mitochondrial pathway in myeloid leukemic cells. *Cell Cycle*. **2003**, 2, 488–493.

3. Shinohara, E.T.; Hallahan, D.E.; Lu, B. The use of antisense oligonucleotides in evaluating survivin as a therapeutic target for radiation sensitization in lung cancer. *Biol Proced Online*. **2004**, 6, 250–256.

4. Lu, B.; Mu, Y.; Cao, C.; Zeng, F.; Schneider, S.; Tan, J.; Price, J.; Chen, J.; Freeman, M.; Hallahan, D.E. Survivin as a therapeutic target for radiation sensitization in lung cancer. *Cancer Res*. **2004**, 64, 2840-2845.

5. Ansell, S.M.; Arendt, B.K.; Grote, D.M.; Jelinek, D.F.; Novak, A.J.; Wellik, L.E.; Remstein, E.D.; Bennett, C.F.; Fielding, A. Inhibition of survivin expression suppresses the growth of aggressive non-Hodgkin's lymphoma. *Leukemia*. **2004**, 18, 616-623.

6. Hansen, J.B.; Fisker, N.; Westergaard, M.; Kjaerulff, L.S.; Hansen, H.F.; Thrue, C.A.; Rosenbohm, C.; Wissenbach, M.; Orum, H.; Koch, T. SPC3042: a proapoptotic survivin inhibitor. *Mol Cancer Ther*. **2008**, 7, 2736-2745.

7. Talbot, D.C.; Ranson, M.; Davies, J.; Lahn, M.; Callies, S.; Andre, V.; Kadam, S.; Burgess, M.; Slapak, C.; Olsen, A.L.; et al. Tumor survivin is downregulated by the antisense oligonucleotide LY2181308: a proof-of-concept, first-in-human dose study. *Clin Cancer Res*. **2010**,16, 6150–6158.

8. Carrasco, R.A.; Stamm, N.B.; Marcusson, E.; Sandusky, G.; Iversen, P.; Patel, B.K. Antisense inhibition of survivin expression as a cancer therapeutic. *Mol Cancer Ther*. **2011**, 10, 221–232.

9. Tanioka, M.; Nokihara, H.; Yamamoto, N.; Yamada, Y.; Yamada, K.; Goto, Y.; Fujimoto, T.; Sekiguchi, R.; Uenaka, K.; Callies, S.; et al. Phase I study of LY2181308, an antisense oligonucleotide against survivin, in patients with advanced solid tumors. *Cancer Chemother Pharmacol*. **2011**, 68, 505–511.

10. Olsen, A.L.; Davies, J.M.; Medley, L.; Breen, D.; Talbot, D.C.; McHugh, P.J. Quantitative analysis of survivin protein expression and its therapeutic depletion by an antisense oligonucleotide in human lung tumors. *Mol Ther Nucleic Acids*. **2012**, 1, e30.

11. Erba, H.P.; Sayar, H.; Juckett, M.; Lahn, M.; Andre, V.; Callies, S.; Schmidt, S.; Kadam, S.; Brandt, J.T.; Van Bockstaele, D.; et al. Safety and pharmacokinetics of the antisense oligonucleotide (ASO) LY2181308 as a single-agent or in combination with idarubicin and cytarabine in patients with refractory or relapsed acute myeloid leukemia (AML). *Investig New Drugs*. **2013**, 31, 1023–1034.

12. Natale, R.; Blackhall, F.; Kowalski, D.; Ramlau, R.; Bepler, G.; Grossi, F.; Lerchenmuller, C.; Pinder-Schenck, M.; Mezger, J.; Danson, S.; et al. Evaluation of antitumor activity using change in tumor size of the survivin antisense oligonucleotide LY2181308 in combination with docetaxel for second-line treatment of patients with non-small-cell lung cancer: a randomized open-label phase II study. *J Thorac Oncol*. **2014**, 9, 1704–1708.

13. Wiechno, P.; Somer, B.G.; Mellado, B.; Chlosta, P.L.; Cervera Grau, J.M.; Castellano, D.; Reuter, C.; Stockle, M.; Kamradt, J.; Pikiel, J.; et al. A randomised phase 2 study combining LY2181308 sodium (survivin antisense


[OFFICIAL]

oligonucleotide) with first-line docetaxel/prednisone in patients with castration-resistant prostate cancer. *Eur Urol*. **2014**, 65, 516–520.

14. Zhao, X.; Xu, J.; Liang, X.; Wang, Z.; Zhu, Y.; Guo, D.; Wang, J.; Amu, G.; Wang, Q.; Yang, Z.; Tang, X. NQO1-activatable circular antisense oligonucleotides for tumor-cell-specific survivin gene silencing and antitumor therapy. *J Med Chem*. **2025**, 68, 4466-4476.

15. Chen, J.; Wu, W.; Tahir, S.K.; Kroeger, P.E.; Rosenberg, S.H.; Cowsert, L.M.; Bennett, F.; Krajewski, S.; Krajewska, M.; Welsh, K.; Reed, J.C.; Ng, S.C. Down-regulation of survivin by antisense oligonucleotides increases apoptosis, inhibits cytokinesis and anchorage-independent growth. *Neoplasia*. **2000**, 2, 235-241.

16. Sapra, P.; Wang, M.; Bandaru, R.; Zhao, H.; Greenberger, L.M.; Horak, I.D. Down-modulation of survivin expression and inhibition of tumor growth in vivo by EZN-3042, a locked nucleic acid antisense oligonucleotide. *Nucleosides Nucleotides Nucleic Acids*. **2010**, 29, 97-112.

17. Lamers, F.; van der Ploeg, I.; Schild, L.; Ebus, M.E.; Koster, J.; Hansen, B.R.; Koch, T.; Versteeg, R.; Caron, H.N.; Molenaar, J.J. Knockdown of survivin (BIRC5) causes apoptosis in neuroblastoma via mitotic catastrophe. *Endocr Relat Cancer*. **2011**, 18, 657-668.

18. Park, E.; Gang, E.J.; Hsieh, Y.T.; Schaefer, P.; Chae, S.; Klemm, L.; Huantes, S.; Loh, M.; Conway, E.M.; Kang, E.S.; Hoe Koo, H.; Hofmann, W.K.; Heisterkamp, N.; Pelus, L.; Keerthivasan, G.; Crispino, J.; Kahn, M.; Müschen, M.; Kim, Y.M. Targeting survivin overcomes drug resistance in acute lymphoblastic leukemia. *Blood*. **2011**, 118, 2191-2199.

19. Morrison, D.J.; Hogan, L.E.; Condos, G.; Bhatla, T.; Germino, N.; Moskowitz, N.P.; Lee, L.; Bhojwani, D.; Horton, T.M.; Belitskaya-Levy, I.; Greenberger, L.M.; Horak, I.D.; Grupp, S.A.; Teachey, D.T.; Raetz, E.A.; Carroll, W.L. Endogenous knockdown of survivin improves chemotherapeutic response in ALL models. *Leukemia*. **2012**, 26, 271-279.

20. Raetz, E.A.; Morrison, D.; Romanos-Sirakis, E.; Gaynon, P.; Sposto, R.; Bhojwani, D.; Bostrom, B.C.; Brown, P.; Eckroth, E.; Cassar, J.; et al. A phase I study of EZN-3042, a novel survivin messenger ribonucleic acid (mRNA) antagonist, administered in combination with chemotherapy in children with relapsed acute lymphoblastic leukemia (ALL): a report from the therapeutic advances in childhood leukemia and lymphoma (TACL) consortium. *J Pediatr Hematol Oncol*. **2014**, 36, 458–463.

21. Thamm, D.H.; Joseph, J.K.; Rose, B.J.; Meuten, T.K.; Weishaar, K.M. Phase-I trial of survivin inhibition with EZN-3042 in dogs with spontaneous lymphoma. *BMC Vet Res*. **2020**, 16, 97.

22. Olie, R.A.; Simões-Wüst, A.P.; Baumann, B.; Leech, S.H.; Fabbro, D.; Stahel, R.A.; Zangemeister-Wittke, U. A novel antisense oligonucleotide targeting survivin expression induces apoptosis and sensitizes lung cancer cells to chemotherapy. *Cancer Res*. **2000**, 60, 2805-2809.

23. Dai, D.J.; Lu, C.D.; Lai, R.Y.; Guo, J.M.; Meng, H.; Chen, W.S.; Gu, J. Survivin antisense compound inhibits proliferation and promotes apoptosis in liver cancer cells. *World J Gastroenterol*. **2005**, 11, 193-199.

24. Wu, Y.F.; Liang, X.J.; Liu, Y.Y.; Gong, W.; Liu, J.X.; Wang, X.P.; Zhuang, Z.Q.; Guo, Y.; Shen, H.Y. Antisense oligonucleotide targeting survivin inhibits growth by inducing apoptosis in human osteosarcoma cells MG-63. *Neoplasma*. **2010**, 57, 501-506.

[OFFICIAL]

25. Coma, S.; Noe, V.; Lavarino, C.; Adán, J.; Rivas, M.; López-Matas, M.; Pagan, R.; Mitjans, F.; Vilaró, S.; Piulats, J.; Ciudad, C.J. Use of siRNAs and antisense oligonucleotides against survivin RNA to inhibit steps leading to tumor angiogenesis. *Oligonucleotides*. **2004**, 14, 100-113.

26. Sun, Y.; Lin, R.; Dai, J.; Jin, D.; Wang, S.Q. Suppression of tumor growth using antisense oligonucleotide against survivin in an orthotopic transplant model of human hepatocellular carcinoma in nude mice. *Oligonucleotides*. **2006**, 16, 365-374.

27. Li, Y.; Chen, S.; Rahimizadeh, K.; Zhang, Z.; Veedu, R.N. Inhibition of survivin by 2'-O-methyl phosphorothioate-modified steric-blocking antisense oligonucleotides. *RSC Adv*. **2024**, 14, 13336-13341.

[OFFICIAL]
